# Charting the Path of the Deadly Ebola Virus in Central Africa

**DOI:** 10.1371/journal.pbio.0030403

**Published:** 2005-10-25

Thanks to sensationalized accounts of patients with liquefying flesh and spouting blood, the Ebola virus may well be the most feared disease on the planet. But the reality of the virus, which strikes humans and other primates, is grim enough, with patients experiencing sudden onset of fever, headache, intense weakness, and muscle pain, followed by diarrhea, vomiting, severe rash, organ failure, and massive hemorrhaging, sometimes external, within two to 21 days of exposure. The first human Ebola outbreaks occurred between 1976 and 1979 in Sudan and Zaire (now the Democratic Republic of Congo), where 88% of the 318 infected persons died—a typical mortality rate for this strain, called the Zaire strain of Ebola virus (ZEBOV). It's thought that humans acquired the virus after handling infected gorilla and chimp carcasses.

Over the past ten years, separate outbreaks of the deadly Zaire strain have killed hundreds of humans and tens of thousands of great apes in Gabon and the Republic of Congo—which harbor roughly 80% of the last remaining wild gorilla and chimpanzee populations. Between 1983 and 2000, poaching and logging precipitated catastrophic declines in these great apes, but scientists fear that Ebola may pose an equally deadly threat. Any efforts to contain the next epidemic depend on understanding the dynamics of the virus's spread. [Fig pbio-0030403-g001]

**Figure pbio-0030403-g001:**
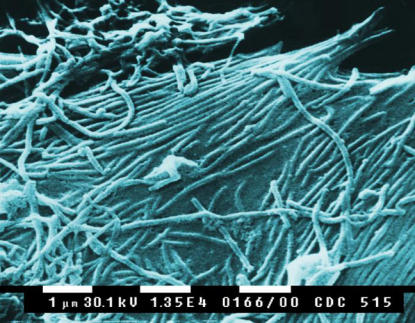
Repeated outbreaks of the Zaire strain of the Ebola virus (Ebola virions pictured above) in central Africa were caused by a recent spread of the virus, rather than by a long-persistent strain at each site

In a new study, Peter Walsh, Roman Biek, and Leslie Real combined genetic data with information on the timing and location of past ZEBOV outbreaks to determine the merits of two competing hypotheses to explain the emergence and spread of the virus. In the prevailing view, ZEBOV arose from long-persistent local strains after increased contact between humans or great apes and an unidentified reservoir host. But Walsh et al. found support for the alternative hypothesis: that ZEBOV had recently spread to the outbreak regions. This is good news because a virus that spreads at a predictable rate in a predictable direction is far easier to control than one that emerges by chance or at the hands of an unknown trigger.

The authors modeled the virus's spread based on assumptions of a long-persistent virus versus a recently emerged virus, and tested the predictions of these competing hypotheses using genetic data—gathered from gene sequences taken from human samples at the different outbreak sites—and information on the spatiotemporal dynamics of the outbreaks. Charting the evolutionary relationships of the viral genotypes identified one major lineage with a most recent common ancestor consistent with the 1976 outbreak. Comparing the path of descent with outbreak localities mirrored the timing of the outbreaks, with new outbreaks directly descending from those preceding.

Analyzing the spatiotemporal pattern of outbreaks revealed a spread at the rate of about 50 kilometers/year—a predictable path not likely for a persistent virus—with the first epidemic in Yambuku, then spreading south to Kikwit and west to Booué, Gabon. Plotting the geographic distribution of genotypes revealed a clear spatial structure at both local and regional scales: the genotypes from the 2001–2003 Gabon/Congo outbreaks, for example, decreased in genetic similarity as distance increased. Again, this finding is consistent with the recently emerged hypothesis, which predicts a correlation between genotype and geography at different distances. Simulations of viral evolution in a spreading epidemic also showed a consistent spread pattern and a strong correlation between genetic divergence and spatial separation. Though the authors can't say how the virus was transmitted, the simulations showed that a “simple nearest neighbor contact process” could produce the linear, uniform spread rates found here.

Though the strength of the individual lines of evidence—timing of origin, spatial spread, and genetic/distance ratio—is not conclusive when considered separately, taken together, they support the hypothesis that a “consistently moving wave of ZEBOV infection” recently spread to outbreak sites in Gabon and Congo. Following its current course, ZEBOV may hit populated areas east of Odzala National Park within 1–2 years and reach most parks containing large populations of western gorillas in 3–6 years. Two Ebola outbreaks have already hit human populations west of Odzala, and over the past two years, the largest gorilla and chimp populations in the world, found in Odzala, have been devastated—the disease is spreading to the last unaffected sector of the park right now. These findings suggest that strategies to protect villagers and some of the last remaining wild apes from future outbreaks would do best to concentrate efforts at the front of the advancing wave—and start acting now. —*Liza Gross*

